# Epileptic foci localization based on mapping the synchronization of dynamic brain network

**DOI:** 10.1186/s12911-019-0737-8

**Published:** 2019-01-31

**Authors:** Tian Mei, Xiaoyan Wei, Ziyi Chen, Xianghua Tian, Nan Dong, Dongmei Li, Yi Zhou

**Affiliations:** 1 Department of Biomedical Engineering, Zhongshan School of Medicine, Sun Yat-sen University, Guangzhou, 510080 China; 2 Department of Information, Sixth Affiliated Hospital, Sun Yat-sen University, Guangzhou, 510655 China; 3 Department of Neurology, First Affiliated Hospital, Sun Yat-sen University, Guangzhou, 510080 China; 4 Department of Medical Engineering and Technology, Xinjiang Medical University, Urumqi, 830011 China; 5 College of Public Health, Xinjiang Medical University, Urumqi, 830011 China

**Keywords:** Epilepsy, Synchronization, Dynamic brain network, Foci location

## Abstract

**Background:**

Characterizing the synchronous changes of epileptic seizures in different stages between different regions is profound to understand the transmission pathways of epileptic brain network and epileptogenic foci. There is currently no adequate quantitative calculation method for describing the propagation pathways of electroencephalogram (EEG) signals in the brain network from the short and long term. The goal of this study is to explore the innovative method to locate epileptic foci, mapping synchronization in the brain networks based on EEG.

**Methods:**

Mutual information was used to analyze the short-term synchronization in the full electrodes; while nonlinear dynamics quantifies the statistical independencies in the long –term among all electrodes. Then graph theory based on the complex network was employed to construct a dynamic brain network for epilepsy patients when they were awake, asleep and in seizure, analyzing the changing topology indexes.

**Results:**

Epileptic network achieved a high degree of nonlinear synchronization compared to awake time. and the main path of epileptiform activity was revealed by searching core nodes. The core nodes of the brain network were in connection with the onset zone. Seizures always happened with a high degree of distribution.

**Conclusions:**

This study indicated the path of EEG synchronous propagation in seizures, and core nodes could locate the epileptic foci accurately in some epileptic patients.

## Background

Epilepsy is an abnormal disease of the brain network [1], which is caused by large super-synchronous neuronal discharge [2]. The synchronization between different brain regions infers the dynamical interactions of segregated brain regions [3]. Characterizing the synchronous changes of epileptic seizures in different stages and investigating the propagation of electroencephalogram (EEG) signals in the brain network will be profound to understand the transmission pathways of epileptic brain network and locate the seizure onset zone [4, 5].

Given the nature of epilepsy, there are undeniably theoretical hurdles to investigate signal synchronization widely, including linear and nonlinear methods [6]. Mutual information (MI) theory can be used to reveal the internal hidden relationships between synchronous signals in short-term [7, 8]. Nonlinear dynamics theory quantifies the nonlinear dependencies among the dynamics of simultaneously recorded signals in long-term [9, 10]. Studies have shown that nonlinear synchronization can be applied to evaluate the connectivity of cortex functions in different brain regions and for epileptic foci localization in long-term [11]. However, few studies have focused on the combination of short-term and long-term changes to explore synchronous brain networks.

Based on the synchronization, complex network technology is frequently used to investigate the integration of functionally specialized brain regions in a network [12, 13]. Some studies summarized analytical and methodological elements of epileptic network studies and discussed findings from recent detailed electrophysiological investigations [14]. The preliminary study suggested that seizure foci may be the weakest connected regions in the brain at the beginning of a seizure and the most strongly connected regions may be connected towards the end of a seizure [15]. Hao [16] indicated clustering coefficient was statistically higher in the ictal period than in the inter-ictal period, and there is no obvious difference for their average path length. Some other complex network indicators were also applied to brain network analysis [17]. However, the brain network was mostly limited to medical imaging in a static network. Scale EEG signals have the advantages of superior time-space resolution and real-time monitoring, which make it an excellent tool for constructing dynamic brain networks [18, 19].

The study was aimed to construct dynamic brain networks by mapping the synchronization of the nonlinear characteristics of the EEG. The characteristics of the complex network were analyzed, the propagation of epileptic discharges described in the short and long term. This study fills a void in the field of the synchronization relationships of the dynamic brain network, contributing to the localization of seizures.

## Materials and methods

### Data resource and data preparation

#### Data resource

1) Data inclusion criteria.

In order to ensure the brain network construction and avoid artifacts as much as possible, the data were selected by the following criteria:

*a)* Patients with focal neocortical epilepsy.

*b)* Obvious EEG changes with inconspicuous body movements.

*c)* Each patient’s long-range EEG test chart must contain at least six or more seizures.

2) Data details.

Based on the above criteria, we can obtain sufficient repetitive sample data and relatively clean sample data for each patient. Patient1 was a 23-year-old female diagnosed with temporal lobe epilepsy, received treatment at the Department of Neurology, the First Affiliated Hospital of Xinjiang Medical University, Urumqi, China. She had 7 seizures and left temporal postoperative changes detected through MRI. Patient 2 was a 6-year-old boy diagnosed with frontal lobe epilepsy, received treatment at the Department of Neurology, the First Affiliated Hospital of Sun Yat-sen University, Guangzhou, China, He had 15 seizures and focal cortical dysplasia in the right front lobe detected by MRI.

#### Data preparation

Electrodes were placed according to the international 10–20 system. A 24-h video-EEG system was used to collect data for a total of 48 h, with a sampling frequency of 2000 Hz. Then the signal was filtered from 0.3-75 Hz. Other artifacts were removed, which had no apparent behavior during seizures. Each seizure was divided into 2–6 segments. Different size of time window was partitioned into the same overlapping frames and the synchronization was computed.

### Calculation of EEG synchronization

### Calculation of the MIs

Kinney JB et al. defined information from the perspective of information uncertainty [20]. The MI can reflect the magnitude and interdependence of information transmission between the two signals. The lower the MI between the two signals, the less similar information there is between them. When the two signals are independent, the value of MI is zero. By measuring the joint probability density distribution between two variants, MI quantifies the mutual dependence. The algorithm for the calculation of MI is as follows:

Define the probability of discrete random variables *X* and *Y* locating in *N* × *N* regions. If P_i_ represents the probability that x_i_ will occur, then the probability of event {X = x_i_, Y = y_i_} is p_ij_. The joint of the two variables is:

1 $$ {\mathrm{H}}_{\mathrm{xy}}=-{\sum}_{\mathrm{i},\mathrm{j}}^{\mathrm{M}}{\mathrm{p}}_{\mathrm{i}\mathrm{j}}\lg {\mathrm{p}}_{\mathrm{i}\mathrm{j}} $$

The MI of *X* and *Y* is defined as:

2 $$ {\mathrm{I}}_{\mathrm{x}\mathrm{y}}={\mathrm{H}}_{\mathrm{x}}+{\mathrm{H}}_{\mathrm{y}}-{\mathrm{H}}_{\mathrm{x}\mathrm{y}} $$

This study employed the statistical parameters of the total channels MIs, namely the mean value and variable coefficient to describe information interaction and synchronization in the brain.

#### Nonlinear characteristic T-index matrices

The synchronization of nonlinear characteristics between two channels can be described using the T-index [21]. In statistics, a t-test of two independent samples can measure whether the samples have the same distribution. When the distribution of the two samples varies greatly, there will be more correspondingly grouped t-test statistics. In this study, T-index matrices were employed to measure the variation in synchronization of the multi-nonlinear characteristics of all channels.

Algorithm for the T-index of every two channels is:

When the number of channels is *N*, the T-index matrix is a *N* × *N* matrix.

3 $$ \mathrm{T}=\frac{\overline{{\mathrm{X}}_1}-\overline{{\mathrm{X}}_2}}{\sqrt{{\mathrm{S}}_{\mathrm{C}}^2\left(\frac{1}{{\mathrm{n}}_1}+\frac{1}{{\mathrm{n}}_2}\right)}} $$

4 $$ {\mathrm{S}}_{\mathrm{C}}^2=\frac{{\mathrm{S}}_1^2\left({\mathrm{n}}_1-1\right)+{\mathrm{S}}_2^2\left({\mathrm{n}}_2-1\right)}{{\mathrm{n}}_1+{\mathrm{n}}_2-2} $$

$\overline{{\mathrm{X}}_1}\mathrm{and}\overline{{\mathrm{X}}_2}$ are the mean values of the two samples, ${\mathrm{S}}_{\mathrm{C}}^2$ is the standard deviation of the difference of the mean values, ${\mathrm{S}}_1^2{\mathrm{andS}}_2^2$ are the variances of the two samples. n_1_andn_2_ are the data lengths of the samples.

#### Calculation of nonlinear characteristics

By reconstructing the phase space, Correlation dimension(CD), approximate entropy(ApEn), Hurst exponent (HE), and the principal component analysis index (PCAI) [22, 23] were calculated to describe the chaotic EEG signals.

#### Cd

CD is a fractal dimension used for the quantitative description of the self-similar structure of a chaotic attractor [24]. This algorithm uses the time series to calculate CD directly, which is by far the most common method. Here, the embedding dimensions for the reconstruction of phase space were calculated using improved Cao method [25] using the nearest neighbors. The delay time was calculated using mutual information. The CD algorithm is as follows:

Calculate the contextual integral of the oscillators:

5 $$ \mathrm{C}\left(\mathrm{r}\right)=\frac{2}{{\mathrm{N}}_{\mathrm{m}}\left({\mathrm{N}}_{\mathrm{m}}-1\right)}{\sum}_{\mathrm{i}=1}^{{\mathrm{N}}_{\mathrm{m}}}{\sum}_{\begin{array}{c}\mathrm{j}=1\\ {}\mathrm{j}\ne \mathrm{i}\end{array}}^{{\mathrm{N}}_{\mathrm{m}}}\mathrm{H}\left(\mathrm{r}-{\mathrm{r}}_{\mathrm{i}\mathrm{j}}\right) $$

*C*(*r*) is the correlation integral function of the signals, where *r* is the hypersphere radius in phase space andm is the embedding dimension. r_ij_ = ‖x(i) − x(j)‖ represents the Euclidean distance between two random points x(i) and x(j) among the N_m_ sample points, among which $$ H(r)=\left\{\begin{array}{c}0,r\le 0\\ {}1,r>0\end{array}\right. $$ is the Heaviside function.

Within a certain range, C(r) ∝ r^D(m)^(r → 0), when N →  ∞ , D(m) is the correlation dimension:

6 $$ {\mathrm{d}}_2=\underset{\mathrm{r}\to 0}{\lim}\frac{\ln \mathrm{C}\left(\mathrm{r}\right)}{\ln \mathrm{r}} $$

Draw scale curve lnr − ln C(r), the straight part of which will be the scaling region. Fit the straight line through least squares method. The straight slope is the CD.

#### ApEn

Approximate entropy is a non-dimensional parameter to represent a signal character from the perspective of measuring the complexity and regularity of signal sequences. As ApEn measures the probability of new patterns, it can quantitatively describe the information included in the specific sequence. The algorithm is described in our previously published work [26]. Here we set the embedding dimension of compared sequences to *m* = 2, the threshold value to *r*=0.25, and the unit time duration for calculation to 1 s.

#### He

The Hurst exponent is a statistical parameter used to assess the chaotic characteristics of time sequences. Which could accurately reveal tendencies in time sequences. The value of HE is between 0 and 1, and quantitatively reflects the long-range correlation between sequences. Rescaled range (R/S) analysis [27].

The algorithm is as follows:

For a given time sequence {x(i)| i = 1, 2, ⋯N}, define the average error of the first *k* points as:

7 $$ {\mathrm{W}}_{\mathrm{k}}={\mathrm{x}}_1+{\mathrm{x}}_2+\cdots +\mathrm{k}\overline{\mathrm{x}}\left(\mathrm{n}\right) $$

In the above formula, $\overline{\mathrm{x}}\left(\mathrm{n}\right)$ is the average value of*x*_1_(*i* = 1, 2 … *N*), 1 ≤ *k* ≤ *n*, 1 ≤ *n* ≤ *N*.

Calculate the difference between the minimum and maximum value of *n*’s correspondent average error:

R(n) = max(W_1_ … W_n_) − min(W_1_ … W_n_), n = 1,2....N (8)

9 $$ \frac{\mathrm{R}\left(\mathrm{n}\right)}{\mathrm{S}\left(\mathrm{n}\right)}=\frac{\max \left({\mathrm{W}}_1\dots {\mathrm{W}}_{\mathrm{n}}\right)-\min \left({\mathrm{W}}_1\dots {\mathrm{W}}_{\mathrm{n}}\right)}{\sqrt{{\mathrm{S}}^2\left(\mathrm{n}\right)}} $$

In the above formula, S(n) is the standard deviation of xi(i= 1,2…N)..

Transform the above equation:

$$ \frac{\mathrm{R}\left(\mathrm{n}\right)}{\mathrm{S}\left(\mathrm{n}\right)}=\mathrm{a}\times {\mathrm{n}}^{\mathrm{H}},\mathrm{n}=1,2\dots $$N (10).

In the above formula, *H* represents the Hurst exponent, then *H* is:

$$ \mathrm{H}\left(\mathrm{n}\right)=\frac{\log \left[\raisebox{1ex}{$\mathrm{R}\left(\mathrm{n}\right)$}\!\left/ \!\raisebox{-1ex}{$\mathrm{S}\left(\mathrm{n}\right)$}\right.\right]}{\log \left(\mathrm{n}\right)},\mathrm{n}=1,2\dots $$N (11).

#### PCAI

PCA is a statistical approach for character extraction [28]. Principal component analysis (PCA) was used to represent integrated characteristics and represent the center of gravity position of data points and the dispersion range of group points. In this study, the PCAI represents the distribution balance of nonlinear eigenvectors extracted through PCA. The algorithm is as follows:

Calculate the average value *E*(*x*) of each row in reconstructed phase space matrix *Y*, and calculate the difference between elements in each row of the phase space matrix and the average value of this row, namely

12 $$ Y=X-E(x). $$

Calculate the covariance matrix:

13 $$ \mathrm{A}=\frac{\left({\mathrm{Y}}^{\mathrm{T}}\mathrm{Y}\right)}{\mathrm{n}-\left(\mathrm{m}-1\right)\uptau} $$

Calculate the *A’*s eigenvalue p_i_ and its correspondent eigenvector *Ui* (*i* = 1, 2, …*m*). The eigenvalue and eigenvector are the principal components.

Sum all the eigenvalues:

14 $$ \upgamma =\sum \limits_{\mathrm{i}=1}^{\mathrm{m}}{\mathrm{p}}_{\mathrm{i}} $$

Then range the eigenvectors according to their values from small to large. Calculate the standard deviation of principal components distribution:

*PCAI=STD(log(*p_i_*/*γ*))* (15).

### Construction of brain network

#### The rule for construction network

MI and nonlinear indexes of pairs of nodes were first calculated. The average MI of all channels was selected as the threshold. When two channels’ MIs was larger than the threshold of the total channels, the functions of these two brain regions were considered to be functionally related. and brain network could be constructed. Then T-matric index of nonlinear characteristic was applied based on such a model.

#### Methods of localizing core nodes

Degree distribution was chosen as the index for measuring network synchronization [29]. The node with the biggest degree is defined as the central node which plays an important role in communication within a brain. If the number of nodes linked to node *i* is *k*(*i*), then its degree is *k*(*i*). Nodes with the largest degrees were marked as the key nodes and the pathways between them marked as key pathways. The actual anatomical connectivity between the two regions was not considered in the determination of correlation.

#### Statistical analysis

Statistics were performed using analysis of variance (ANOVA) models to examine the different characteristic values of synchronization, if data had normal distributions and homogeneous variances. Otherwise, the Kruskal-Wallis test (K-W test) for nonparametric statistics was employed. The Tukey method, with its high degree of stability, was used for multiple comparisons. The covariance structure was assumed to be compound and symmetric. Analyses were implemented in SPSS (version 18) with a significance level of 0.05. All data are given as mean ± standard deviation (SD).

## Results

### EEG synchronization in different stages

#### EEG synchronization based on MIs

There were certain differences between the three stages for each patient from the statistical analysis indicated in Tables 1 and 2. For a patient with temporal lobe epilepsy, EEG synchronization is at the highest level in the ictal stage and at its lowest level in the awake stage. The synchronization distribution difference is most unbalanced in the sleep stage and is relatively consistent in the ictal and awake stages. For a patient with frontal lobe epilepsy, EEG synchronization is at the highest level in the ictal stage and synchronization distribution difference is most unbalanced in the ictal stage and most consistent in the awake stage.

**Table 1:** Global mutual information in three stages

EEG	Mean		Coefficient of Variation	
	TLE	FLE	TLE	FLE
AS	0.926 ± 0.132	1.607 ± 0.066	7.549 ± 2.619	7.960 ± 1.647
SS	1.062 ± 0.062	1.600 ± 0.143	10.201 ± 2.273	9.472 ± 2.108
IS	1.032 ± 0.119	1.771 ± 0.176	8.431 ± 2.206	10.283 ± 1.744

*AS*: Awake stage, *SS*: Sleep stage; *IS*: Ictal stage; *TLE*: temporal lobe epilepsy; *FLE*: frontal lobe epilepsy

**Table 2:** Statistical results of mutual information in three stages

EEG	Mean		Coefficient of Variation	
	TLE	FLE	TLE	FLE
AS and SS	*P* < 0.05*	*P* > 0.05	*P* < 0.05*	*P* < 0.05*
AS and IS	*P* < 0.05*	*P* < 0.05*	*P* > 0.05	*P* < 0.05*
SS and IS	*P* > 0.05	*P* < 0.05*	*P* < 0.05*	*P* < 0.05*
ANOVA	–	–	*P* < 0.05*	–
K-W test	*P* < 0.05*	*P* < 0.05*	–	*P* < 0.05*

*AS*: Awake stage; *SS*: Sleep stage; *IS*: Ictal stage; *TLE*: temporal lobe epilepsy; *FLE*: frontal lobe epilepsy

#### EEG synchronization based on nonlinear dynamics

Table 3 shows T-index averages in different stages under global channels. Table 4 displays the corresponding T-test. The EEG of Patient 1, with temporal lobe epilepsy, revealed the prominent statistical difference between the awake and ictal stages. Compared with the awake stage, the ictal stage had a lower synchronization level of ApEn and HE and a higher degree of CD (*p* < 0.05). The statistical difference between the awake stage and the ictal stage only manifested in the T matrix of HE (*p* < 0.05).

**Table 3:** the T-index average value of nonlinear eigenvalue in three stages

stage	CD		ApEn		Hurst		PCAI	
	TLE	FLE	TLE	FLE	TLE	FLE	TLE	FLE
AS	1.452	3.902	3.220	2.070	3.099	1.262	1.816	1.075
SS	1.843	3.244	1.948	5.317	2.479	3.194	1.839	1.873
IS	2.154	3.801	1.913	4.443	1.395	2.301	1.601	3.950

*AS:* Awake stage; *SS*: Sleep stage; *IS*: Ictal stage; *TLE*: temporal lobe epilepsy; *FLE*: frontal lobe epilepsy

**Table 4:** Statistical results of T-index of nonlinear eigenvalues in three stages

stage	CD		ApEn		Hurst		PCAI	
	TLE	FLE	TLE	FLE	TLE	FLE	TLE	FLE
AS and SS	*P* < 0.05*	*P* < 0.05*	*P* < 0.05*	*P* < 0.05*	*P* > 0.05	*P* < 0.05*	*P* > 0.05	*P* < 0.05*
AS and IS	*P* < 0.05*	*P* < 0.05*	*P* < 0.05*	*P* < 0.05*	*P* < 0.05*	*P* > 0.05	*P* > 0.05	*P* < 0.05*
SS and IS	*P* > 0.05	*P* > 0.05	*P* > 0.05	*P* < 0.05*	*P* < 0.05*	*P* < 0.05*	*P* > 0.05	*P* < 0.05*
K-W test	*P* < 0.05*	*P* < 0.05*	*P* < 0.05*	*P* < 0.05*	*P* < 0.05*	*P* < 0.05*	*P* > 0.05	*P* < 0.05*

*AS*: Awake stage; *SS*: Sleep stage; *IS*: Ictal stage; *TLE*: temporal lobe epilepsy; *FLE*: frontal lobe epilepsy

The EEG of the patient with frontal lobe epilepsy revealed significant differences between PCAI and ApEn in the three stages (*p* < 0.05). Compared with the awake stage, the ictal stage had a higher synchronization level of CD and PCAI and lower ApEn. Statistical differences between the awake and sleep stages were revealed by all of the 4 eigenvalues (*p* < 0.05).

**Fig. 1:**
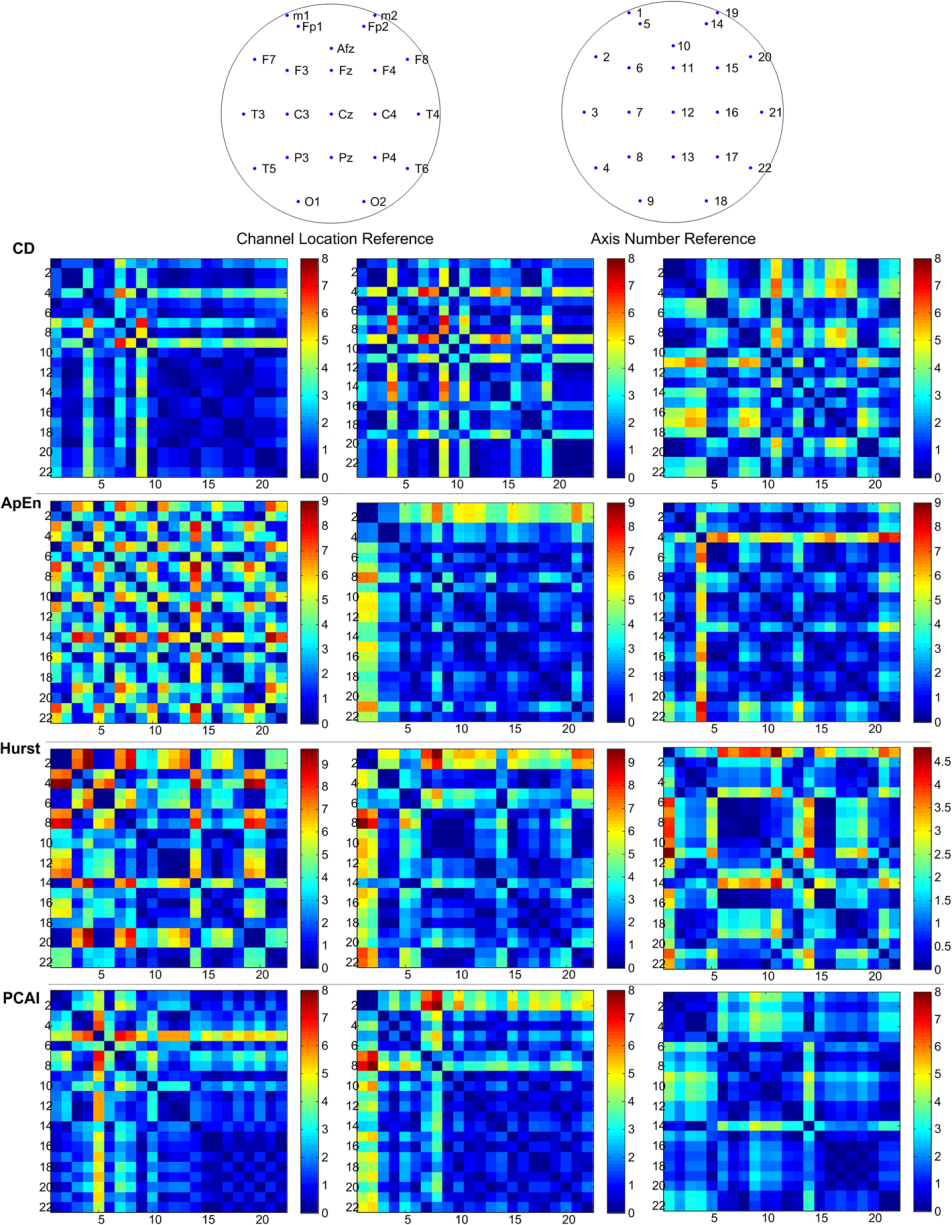
The T-index matrix of nonlinear eigenvalues in temporal lobe epilepsy. The coordinates are channel numbers. Numbers 1–9 correspond to the right hemisphere and numbers 14–22 correspond to the right hemisphere. The CD T-index increases gradually. For ApEn, Hurst, and PCAI, the T-index decreases gradually and ApEn outstands in T5 channel as the color is more vivid

T-index matrix values were distributed differently in different stages. For the patient with temporal lobe epilepsy, the seizure usually happened in the sleep stage. From the awake stage to the sleep stage and the ictal stage, the T-index matrix for the nonlinear eigenvalue gradually changed (see Fig. 1). As the CD revealed, the signals in fractal appear to be progressively more consistent as the color is more balanced. In the awake stage, the right hemisphere EEG is highly consistent, while global activity is highly consistent in the ictal stage and the ApEn, HE, and PCAI synchronization levels declined. The ApEn matrix outstands in T5 channel as the color is more vivid, and the PCAI matrix also outstands in a similar area. In the patient with frontal lobe epilepsy, seizures usually occurred in the sleep stage. From the sleep stage to the awake stage and on to the ictal stage, only the PCAI T-index matrix showed gradual change.

### Dynamic network construction results

#### Pathway of synchronous discharge

**Fig. 2:**
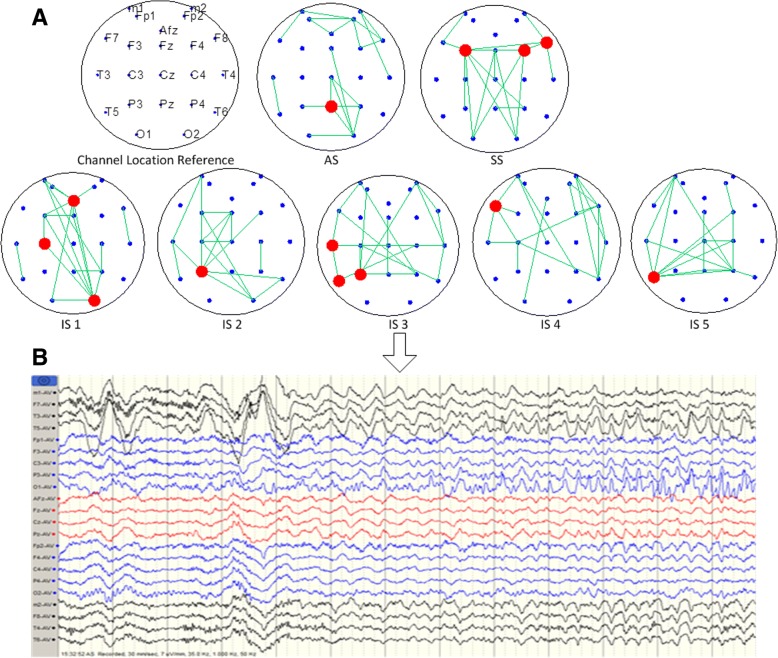
Construction result of EEG brain network based on mutual information in temporal lobe epilepsy. **A** Each figure shows that the connection of brain EEG segments lasts for 12 s. The five figures in the second line show five sequential stages of a seizure. The bold points represent the core node channels. The main path of the network is: Pz-(F3\F4\F8) -(C3\O2)-P3-(T3\P3)-F7-T5. **B** The EEG segment of IS2. The EEG waves start in the left temporal lobe and bursting spikes can be seen in the T3 and T5 channels. The surface channels m1 and m2, which replace deep sphenoid electrodes, are included in the 22 channels. t. AS = Awake stage; SS=Sleep stage; IS=Ictal stage

**Fig. 3:**
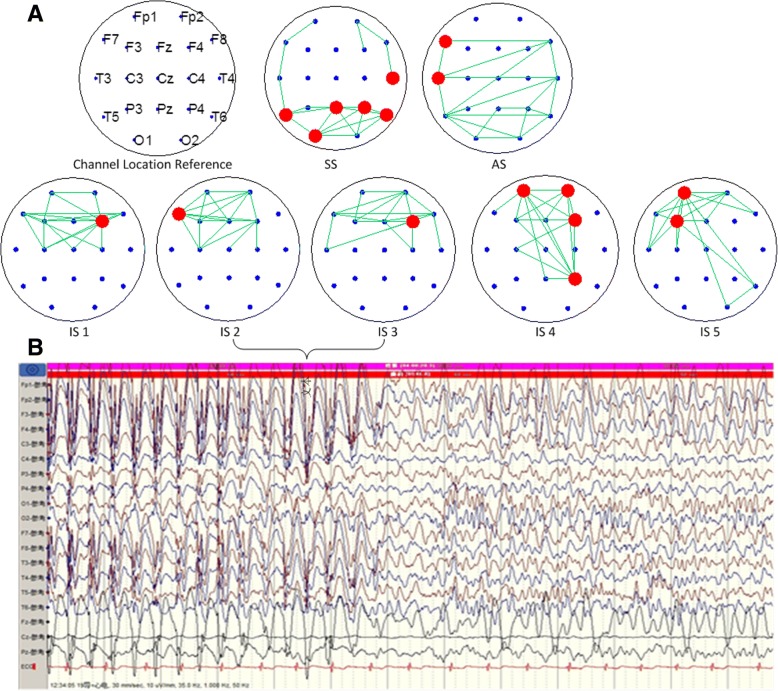
Construction result of EEG brain network based on mutual information in frontal lobe epilepsy. **A** Each figure shows that the connection of brain electrical sections lasts for 6 s. The six figures in the second line show five sequential stages of a seizure. The bold points represent the core node channels. The main path of the network is: (P4\O1\T4\T5) -(F7\T3)-F4-F7-Fz-(Fp1\Fp2\F4) -(Fp1\F3). **B** The EEG segments of IS2 and IS3. Epileptiform discharge in the prefrontal area is observed. The results show spike and wave complexes and poly-spike wave complexes and these mainly occurred in F4 and Fp2

In this study, the maximum number of connections of the constructed brain network was 271. 20 pairs of channels with the largest MI were displayed in the brain network. Each figure has 20 channels, as shown in Fig. 2 and Fig. 3. The core nodes of the MI network indicated the location of the abnormal channel. Similar to the brain function network construction based on MI, network topology with four indexes can easily be constructed. The meaning of this remains unclear.

#### Core node of brain network

The core node in the network is the largest node of the degree distribution. The rubricated channels shown in Fig. 2 and Fig. 3 are brain network core nodes, the information of which is exchanged with the most active nodes with the strongest synchronization impact. From the beginning of sleep stage 2, the core nodes indicate coincide well with the location of an abnormal channel (T3 and T5 for the patient with temporal lobe epilepsy; F4 and Fp2 for the patient with frontal lobe epilepsy). These core nodes near the seizure focus area or appear as a corresponding offside locus.

**Fig. 4:**
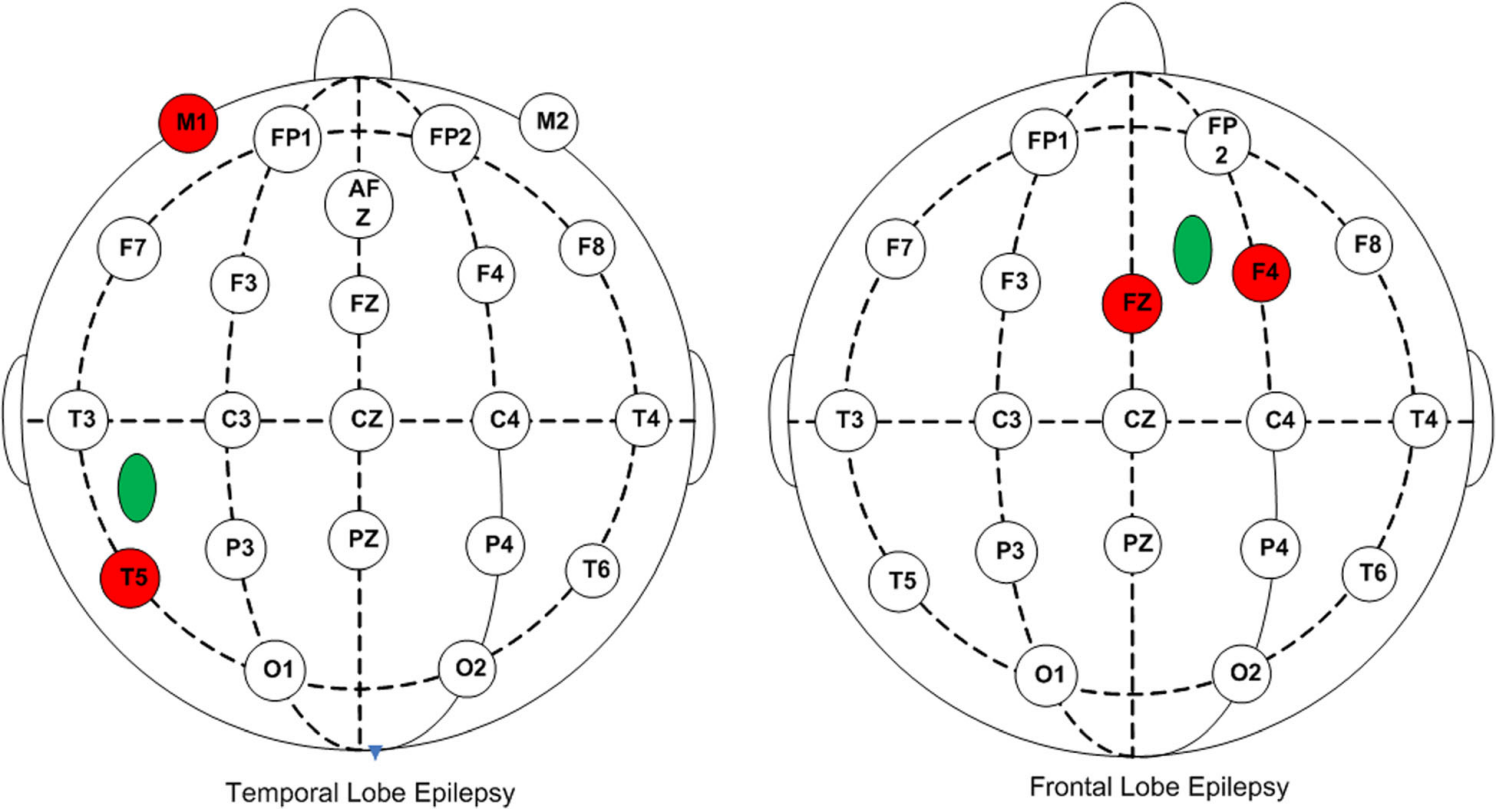
The key channel positions of a brain network constructed based on nonlinear characteristics. Round areas with color are location key channels and elliptic areas with color are seizure foci. The location of the deep sphenoid electrode is represented by the M1 channel

Conversely, the core node of the network with four indexes shows that in the ictal stage, the characteristic values trend towards deviating to the brain hemisphere with the seizure focus. By counting the frequency of the core nodes simultaneously indicated by four characteristic networks, the two channels with the highest node degrees were obtained. As shown in Fig. 4, the network node with nonlinear characteristics in the ictal stage accords with the clinical result of the seizure focus.

#### Degree distribution of brain network

**Fig. 5:**
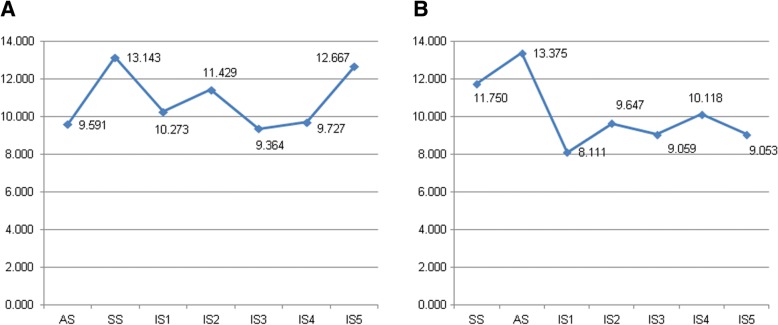
The degree distribution of the brain network in different states. **A** The degree distribution of the brain network in different states in the patient with temporal lobe epilepsy. **B** The degree distribution of the brain network in different states in the patient with frontal lobe epilepsy

The degree distribution of the brain network is shown in Fig. 5. The seizures happen in the brain network state with relatively high degree distribution. When the stage transitions from inter-ictal to seizure, the degree distribution of the whole network shows an initial decrease. During the seizure, the low degree distribution of brain network shows a slight increase and then decreases again.

## Discussion

According to the synchronization, the whole brain synchronization varies with different EEG states. The distribution of MI in different brain regions differs significantly in the same state. Previous studies have revealed that the MI of each channel in the epileptic foci is higher in seizures than interictal state [30]. This study shows that MI synchronization in the epileptic seizure stage is higher than in the awake stage, which is consistent with the global synchronization analysis result obtained by previous studies [31, 32]. Meanwhile, EEG signal synchronization is at the lowest level in the awake stage, which is believed to be a result of increased autonomous brain activity in the awake stage. It is worth noting that the rise of EEG signal synchronization in the sleep stage is very probably due to the high level of synchronization in partial regions.

The awake stage based on nonlinear eigenvalue shows the highest level of ApEn and HE synchronization as well as the lowest degree of CD, while the seizure stage has the highest degree of CD. Both patients had individual discrepancies in PCAI synchronization, which may arise from the different reference electrodes chosen for the channel. Though HE synchronization in both the seizure and sleep stages in the two patients shows discrepancies, the opposite results were observed. This implies that individual discrepancies must be taken into consideration in epileptic seizure detection and the threshold value detection method is unreliable.

The seizures were all found to occur when the brain network has high degrees of distribution. The degrees slightly rise and then drop in the seizure stage. From the perspective of network information flow, epileptic seizures occur more easily when the brain network is highly active. When epileptic seizures occur, the information flow of the brain remains inactive, and the information interaction intensifies as the seizure continues.

For a patient with frontal lobe epilepsy, EEG clinical diagnosis shows that the spike-and-slow wave complex activities are mostly dominated by the right anterior section (F4 and Fp2) and occasionally dominated by the left anterior section (F3 and Fp1) in the seizure stage. These involve the whole channel, and then parts of the seizures shift to anterior-dominated slow activities, involving the temple (F8) and occipital region (O2). Using the network diagram, we can derive that the network in the prefrontal region is active in the seizure stage, with the left and right frontal regions alternately being active, followed by the right posterior occipital region.

For a patient with temporal lobe epilepsy, EEG clinical diagnosis shows that the seizure stage starts from the left temple, and consecutive spike waves can be seen through the left middle and posterior temples (T3 and T5). Using a network diagram, during the seizure the network is first been activated in the left posterior brain region, then becomes symmetrically active in the whole brain, and finally stays active in the right anterior brain region. The brain network connection in the awake stage is quite dispersed. In the sleep stage, temporal lobe epilepsy is active in the frontal region, while in frontal lobe epilepsy activity occurs in the posterior occipital region. In both, activity shies away from the epileptogenic region.

In sum, there are differences between the two patients. For the patient with frontal lobe epilepsy, the main path of the network is: (P4\O1\T4\T5) -(F7\T3)-F4-F7-Fz-(Fp1\Fp2\F4) -(Fp1\F3). While the patient with temporal lobe epilepsy, the main path of the network is: Pz-(F3\F4\F8) -(C3\O2)-P3-(T3\P3)-F7-T5. Contrasted the process of each person’s electroencephalogram to the corresponding clinical diagnosis report, the brain network shifting path in this study is highly consistent with details in the clinical diagnosis report.

In the earlier stage of a seizure, activities of neurons at the seizure focus separate, leading the seizure focus nerves to dissociate from the inhibition of peripheral neurons and discharge with intensity [33]. Previous studies found a difference between the brain graphs of affected and unaffected hemispheres [34, 35] and compared to EEG signals from epileptic brain areas, signals recorded from epileptogenic brain areas are more uniform and nonlinear-dependent [36]. In this study, the epileptic foci are quiet in the interictal periods. However, the epileptogenic region activates in the seizure stage and simultaneously becomes the key node of a brain network. During an epileptic seizure, the brain network core nodes, which are the most active nodes with the strongest synchronization impacts, shift along with the transfer of synchronous discharge [37]. Our results also show that the nonlinear characteristic network nodes are consistent with the localization of epileptic foci during clinical diagnosis. Since this article focus on mapping of the map of the EEG network. The construction method of this paper can also be applied to more samples to explore brain synchronization in different conditions.

## Conclusions

The accurate foci location will revolutionize the internal and surgical treatment of epilepsy. In this study, we have demonstrated that the way synchronization-based brain networks change along with space-time. The path of EEG synchronous propagation in seizures, and core nodes could locate the epileptic foci accurately in some epileptic patients. Especially MI, gives a quantitative information on the degree of information interaction in detail, which can be consistent with the clinical manifestation. Considering that EEG signals reflect the discharges of neurons in the brain, the level of EEG synchronization between channels represents the intensity of information exchange. Therefore, this study may be served as a benchmark for exploring the dynamic brain network. We hope that study will motivate and guide further development of the epileptic network.

## Abbreviations

ANOVA: analysis of variance
ApEn: Approximate Entropy
AS: Awake stage
CD: Correlation Dimension
EEG: electroencephalogram
FLE: frontal lobe epilepsy.
HE: Hurst Exponent
IS: Ictal stage
K-W test: Kruskal-Wallis test
MI: Mutual Information
PCA: Principal Component Analysis
PCAI: Principal Component Analysis Index
SD: standard deviation
SS: Sleep stage
TLE: temporal lobe epilepsy
